# Expression and Secretion of Cyan Fluorescent Protein (CFP) *in B. subtilis* using the Chitinase Promoter from *Bacillus pumilus* SG2

**DOI:** 10.18869/acadpub.ibj.21.4.240

**Published:** 2017-07

**Authors:** Abbas Shali, Garshasb Rigi, Majid Pornour, Gholamreza Ahmadian

**Affiliations:** 1 Department of Industrial and Environmental Biotechnology, National Institute of Genetic Engineering and Biotechnology (NIGEB) , P.O. Box 14155-6343 , Tehran, Iran; 2 Department of Biology, Faculty of Science, Behbahan Khatam Alanbia University of Technology, Behbahan, Khuzestan, Iran; 3 Medical Laser Research Center, Academic Center for Education, Culture and Research (ACECR), Tehran, Iran

**Keywords:** Cyan fluorescent protein, Medical reporter gene, Medical screenable marker, Medicinal protein engineering

## Abstract

**Background:**

Improved cyan fluorescent protein (ICFP) is a monochromic, green fluorescent protein (GFP) derivative produced by *Aequorea macrodactyla* in a process similar to GFP. This protein has strong absorption spectra at wavelengths 426-446 nm. ICFP can be used in cell, organelle or intracellular protein labeling, investigating the protein-protein interactions as well as assessing the promoter activities.

**Methods:**

In our previous study, the promoters of two chitinases (ChiS and ChiL) from *Bacillus pumilus* SG2 were assessed in *B. subtilis* and their regulatory elements were characterized. In the present study, *icfp* was cloned downstream of several truncated promoters obtained in the former study, and ICFP expression was evaluated in *B. subtilis*.

**Results:**

Extracellular expression and secretion of ICFP were analyzed under the control of different truncated versions of ChiSL promoters grown on different media. Results from SDS-PAGE and fluorimetric analyses showed that there were different expression rates of CFP; however, the UPChi-ICFP3 construct exhibited a higher level of expression and secretion in the culture medium.

**Conclusion:**

Our presented results revealed that inserting this truncated form of Chi promoter upstream of the ICFP, as a reporter gene, in *B. subtilis* led to an approximately ten fold increase in ICFP expression.

## INTRODUCTION

Over the past two decades, remarkable progress has been made in the field of viral and non-viral gene transfer systems. It is well known that the measurements of *in vivo* gene expression at different time intervals and gene localization play essential roles in this process[1-3]. The employment of an efficient promoter is one of the most important factors for successful gene expression because of its vital effects on gene transcription both quantitatively and qualitatively.

Numerous promoters have already been isolated from the genomes of different organisms, and great attempts have been made to identify the effects of promoter molecular structures on controlling the gene transcription process. As a consequence, some promoters with well-known structures have been identified, which represented a great potential for gene expression, particularly in plant and animal cells[4]. Such promoters are able to control gene transcription and expression in transgenic plants, as well as are able to make improvements in some agricultural features of crops. For instance, the 35S promoter of cauliflower mosaic virus is the most common promoter used for dicotyledonous transgenic plants[4]. It is well-documented that a promoter with the ability to continuously and permanently express a gene of interest plays an important role in engineering plant or animal cells for either gene therapy purposes or increased resistance against pathogens[5]. Furthermore, these promoters are necessary for the expression of reporter genes in transgenic studies[6]. In our previous studies, a salt-tolerant bacterium was isolated from soil of some regions in Iran. Such bacteria indicated to be able to express both ChiS and ChiL chitinases under the control of a strong promoter[7,8]. The activity of this promoter was evaluated in *B. subtilis* as a host organism. The full-length ChiS promoter has been shown to have a limited function in protein expression; However, deletion of direct repeats or catabolite responsive elements (CRE) in the promoter leads to its increased activity[9,10]. Additionally, improved cyan fluorescent protein (ICFP) is a monochromic, green fluorescent protein (GFP) derivative produced by *Aequorea macrodactyla* in a process similar to that of GFP[11-15]. This fluorescent protein exhibits acceptable absorption spectra at wavelengths of 426 to 446 nm and can be used for labeling purposes[16].

The aim of this study was to quantitatively evaluate the strength of the chitinase promoters created in the previous studies. In this light, the ICFP reporter gene was inserted into the downstream of the signal sequence, as well as the full-length and truncated forms of the ChiSL promoter. Furthermore, the ability of promoter to drive the protein expression was determined using fluorometry analysis.

## MATERIALS AND METHODS

### Bacterial strains, plasmids, media, and growth conditions

Table 1 represents bacterial strains and plasmids used in this study. Bacillus Spizizen Salts (BSS) minimal media were used in the presence of glucose or chitin.

**Table 1:** Primers, plasmids and strains used in this study

Name	Sequence (5’→3’)	Reference
Primers	
UpchiCFP-1	AATGGGGAAAGTGCAAAAGCCGCGGATTCAATAGAAAAGGTAAGC	This study
UpchiCFP-2	GCTTACCTTTTCTATTGAATCCGCGGCTTTTGCACTTTCCCCATT	This study
ChiSLF11	GGGG AAGCTT CGCAGATGT CAT TGAAGT	This study
UP-CRE1	GCATATGAAAACTAGAAATGTTGTTGTCTTCAGTGC	This study
UP-CRE2	GCACTGAAGACAACAACATTTCTAGTTTTCATATGC	This study
UP-sig1	CTCCACTCACATATACGGTTGTTGTTGTCTTCAGTGCC	This study
UP-sig2	GGCACTGAAGACAACAACAACCGTATATGTGAGTGGAG	This study
ChiSLF10	GGGCCCGGGTCATCAAGACGCAGATGTC	[9]
CFPR5	GGG GCATGCTTACTTGTACAGCTCGTC	[9]
ChiSR1J	GGGGCATGCGAGCCCACTCTCTCTTTA	[9]
Plasmids	
pDHAFB	amyE, chloramphenicol resistance	Novagen, Darmstadt, Germany
pUPChi-CFP	PchiS_∆394_—ICFP, chloramphenicol resistance	This study
pUPChi-CFP2	PchiS_∆cre_— ICFP , chloramphenicol resistance	This study
pUPChi-CFP3	PchiS_∆cresig_— ICFP , chloramphenicol resistance	This study
pUPChi Δcre		[9]
pUPChi Δcre-sig		[9]
Strains		
*E. coli* Top10	F0{lacIq Tn10 (TetR)} mcrA D(mrr-hsdRMS-mcrBC) U80lacZDM15 DlacX74 recA1 araD139 D(ara-leu)7,697 galU galK rpsL endA1 nupG-	Novagen, Darmstadt, Germany
*B. subtilis* trpaC2	Nonpathogenic, aerobic, endospore-forming, rod-shaped Gram-positive bacterium, commonly found in soil.	Novagen, Darmstadt, Germany

### Bioinformatic analysis of promoters

All the members of the chitinase protein family were retrieved from the UniProt database in order to find different truncated forms of the chitinase promoter in *Bacillus* species[17]. The NCBI database was used to scan the interested chitinase promoters of nine different species from the *Bacillus* genus. The promoters were aligned in BioEdit v7.0.1[18] using ClustalW Multiple Alignment[19]. The same procedure of multiple alignment analysis was carried out through MEGA5[20] matrix to define the ancestral pattern. Subsequently, the phylogenetic tree was constructed using a bootstrap circulation of 1000 to define the degree of homology between the selected strains.

### Recombinant DNA techniques and oligonucleotides

DNA purification, restriction digestion, ligation, and agarose gel electrophoresis, as well as *E. coli* transformation were performed as described by Green and Sambrook and Russell[21]. Enzymes were obtained from Roche (Mannheim, Germany). *B. subtilis* was transformed based on the standard method described by Leskela *et al*.[22]. Table 1 summarizes the oligonucleotides used in this study.

### Plasmids

All plasmids used in this study were constructed using overlapping PCR. PCR was carried out to construct the plasmid pUP-ChiCFP using primers ChiSLF10 and Up-ChiCFP2, and the plasmid pUPChi-2 as a template (~740 bp). Additional PCR was performed using primers Up-ChiCFP1 and CFPR5, and ICFP-Chi as a template (~780 bp). Afterwards the two PCR products were combined in an equal volume, followed by overlapping PCR using primers ChiSLF10 and CFPR5 (~1500 bp). To construct pUPChi-CFP2, the primary PCR (PCR1) was performed with the primers ChiSLF11 and pUp-ChiCFP2, using pUPChi-2 as a template (~725 bp) and the secondary PCR (PCR2) with the primers CFPR5 and pUP-CRE1 using pUPChi2 as a template (~780 bp). The two PCR products were then combined in equal volumes, followed by overlapping PCR using primers ChiSLF11 and CFPR5 (~1500 bp). PCR amplicons were cloned into the shuttle vector pDHAFB between the *Hind*III and *Sph*I restriction sites. To build pUPChi-CFP3 (lacking CRE box+sigma binding site), PCR1 was performed with the primers ChiSLF10 and UP-sig2 and PCR2 with primers ChiSR1J and UP-sig1, both using pUPChiΔcre[9] as a template (~700 and ~780 bp, respectively). These two PCR products were combined in equal volumes, and overlapping PCR was carried out using primers ChiSLF10 and ChiSR1J (~1480 bp). PCR products were cloned into the pDHAFB vector between the *Cfr*9I and *Sph*I restriction sites. All constructs were transformed into *E. coli* Top10 cells, and the positive transformants were selected on Luria-Bertani medium containing ampicillin. Subsequently, the colonies containing recombinant plasmids were transformed into *B. subtilis* trpaC2, and selected on Luria-Bertani medium containing kanamycin (10 µg/ml) after an incubation at 37°C overnight.

### Expression and secretion of improved cyan fluorescent protein

The coding region of ICFP was fused to the full-length and truncated forms of ChiSL. Following the transformation of *B. subtilis* strains with the prepared constructs, the transformants were grown in BSS minimal media in the presence of glucose or chitin. The expression and secretion of ICFP by recombinant *B. subtilis* containing different constructs were confirmed by a change in the color of the medium from brown to green. *B. subtilis* transformed with pUPChi-CFP was grown in three different minimal media, containing the same concentration of glucose along with mannitol, arabinose, or sorbitol; chitin was added to all the media as described earlier. Afterwards, CFP expression and medium color change were checked. It should be mentioned that these sugars lack the ability to inhibit the ChiSL operon through catabolite repression.

### SDS-PAGE analysis

Cells were separated from the growth medium by centrifugation at 5000 ×g at room temperature for 10 min. Medium supernatants were harvested and then concentrated using the addition of TCA (a final concentration of 12% w/v) on ice for an hour. Equal amounts of proteins were loaded onto the SDS-PAGE gels. Subsequently, cell pellets were resuspended in protoplast buffer (20 mM potassium phosphate pH 7.5, 15 mM MgCl_2_, 20% sucrose, and 1 mg lysozyme/ml) and incubated at 37°C for 30 min. The resulting protoplasts were dissolved in SDS sample buffer, boiled for 5 min and analyzed by SDS-PAGE as described previously[21]. Next, proteins were transferred from the polyacrylamide gel to a polyvinylidene fluoride membrane (Roche, Germany [21]. CFP signal was then detected by sequential incubation of the membrane with polyclonal anti-GFP antibodies (Molecular Probes, Leiden, the Netherlands) and anti-rabbit horseradish peroxidase-conjugated IgG antibodies (Amersham Biosciences, Little Chalfont, United Kingdom) based on the manufacturers’ instructions. GFP shares high amino acid sequence homology with CFP, which makes it possible to use anti-GFP antibodies for the detection of CFP[23].

### Fluorimetric analysis of total extracellular proteins

Cells were separated from the growth medium by centrifugation at 5000 ×g at room temperature for 10 min. The extracellular proteins in the supernatant were collected, and protein pellets were stored for further analysis[24]. Samples were analyzed by a fluorimeter (Perkin-Elmer, Boston, USA) using quartz cuvettes (Hellma, Müllheim, Germany). The instrument settings to measure CFP fluorescence were as follows: excitation at 300 nm and emission at 600 nm. During all measurements, the photomultiplier tube voltage was set at 750 V.

## RESULTS

### Comparison of the amino acid sequence of catabolite responsive element from different Bacillus species

**Fig. 1:**
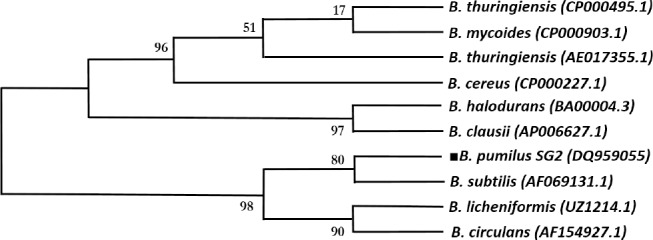
The phylogenetic tree from the chitinase promoter in the *Bacillus* species. The ancestral homology of *B. pumilus* (marked with a black square) is shown through the Bacillus species using original sequence and gene bank accession number of their chitinase promoter.

CRE is considered as one of the fundamental components in promoters of the *B. subtilis* genome. It is also involved in the transcription repression of degradative enzymes in the presence of preferred carbon sources in the *Bacillus* genus. To compare the CRE sequence pattern, the chitinase promoter derived from all *Bacillus* species was selected and aligned using BioEdit program. Nucleotides above the 90% identity were chosen to define a pattern. Following to this procedure, a phylogenetic analysis was performed to define the ancestral homology of *B. pumilus* through the *Bacillus* species using the MEGA5 program (Fig. 1).

**Fig. 2:**
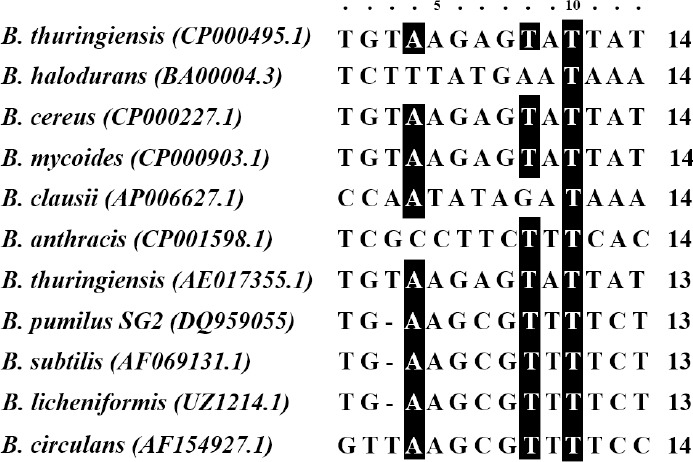
The nucleotide pattern in the *cre* consensus sequence and the number of nucleotides of the chitinase promoter in the *bacillus* species. The black boxes show the identity of the nucleotides.

Comparison of the consensus pattern of CRE in different *Bacillus* species showed that *B. halodurans* and *B. anthracis*, unlike *B. pumilus* SG2, exhibit a rather different pattern. Phylogenetic analysis also demonstrated that *B. pumilus* SG2 shares the most ancestral homology with *B. subtilis* and *B. Licheniformis* (Fig. 2).

### Construction of different promoter variants for catabolic responsive elements expression

**Fig. 3:**
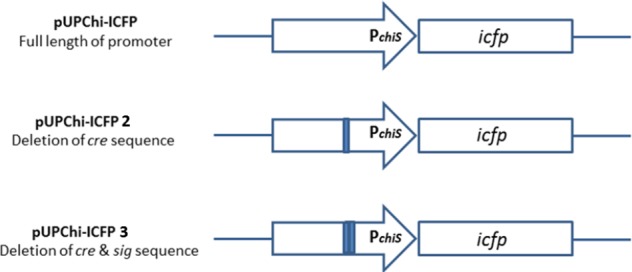
Schematic presentation of the constructs designed to fuse the coding region of *icfp* to the promoter and signal sequence of ChiS.

Fusion of the promoter with an unmodified *icfp* gene in *B. subtilis* has been shown to result in little or no production of fluorescent proteins. For this reason, we attempted to construct improved variants of the promoter expressing ICFP. To this end, the coding sequence of the chitinase promoter from *B. pumilus* SG2 and its variants were fused to the *icfp* gene as a reporter (Fig. 3), and the expression of the reporter gene was evaluated.

### Expression analysis of improved cyan fluorescent protein

**Fig. 4:**
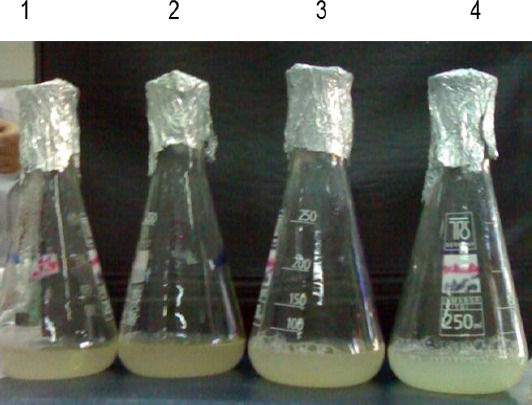
Expression and secretion of ICFP from different truncated versions of the ChiSL promoter. Transformants were inoculated as follows: 1 through 4, *B*. *subtilis* strains transformed with UPChi-ICFP, UPChi-ICFP2, and UPChi-ICFP3 that was induced by glucose as a sole carbon source and UPChi-ICFP3 that was induced by glucose and chitin, respectively.

**Fig. 5:**
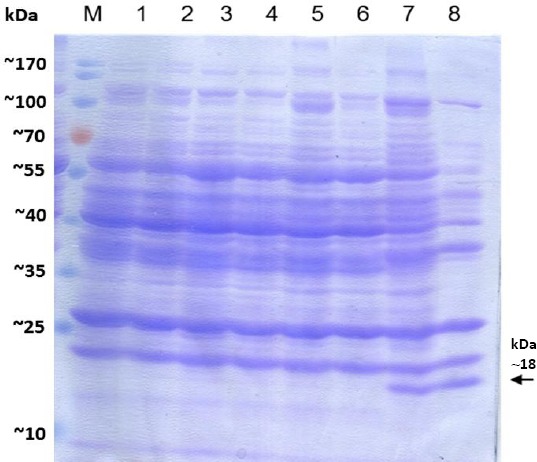
SDS-PAGE analysis of ICFP expression and secretion from different truncated forms of the ChiSL promoter. Lanes 1 and 2, parent *B. subtilis* trpC2 168; lanes 3 and 4, UPChi-ICFP; lanes 5 and 6, UPChi-ICFP2; lanes 7 and 8, *B. subtilis* transformed with UPChi-ICFP3. Lanes 1, 3, 5, and 7 are medium supernatants containing glucose, while lanes 2, 4, 6 and 8 show medium supernatants containing chitin. Lane 8 also indicates a higher expression level. M, stained protein molecular weight marker.

Expression and secretion of ICFP driven by different truncated forms of the ChiSL promoter on minimal media showed that the pUPChi-ICFP3 construct resulted in higher levels of ICFP expression compared to other constructs. This was indicated by color changes in medium (Fig. 4). The SDS-PAGE analysis of culture supernatants obtained from pUPChi-ICFP3 transformants indicated a higher ICFP expression levels in the presence or absence of glucose as a carbon source. Recombinant *B. subtilis* strains transformed with different ICFP constructs were grown in BSS minimal media in the presence of glucose or chitin, or both. SDS-PAGE analysis followed by western blotting did not detect any ICFP protein in bacterial pellets (data not shown). Medium supernatants were concentrated using TCA, and then equal amounts of proteins were subjected to SDS-PAGE and western blot analysis. The results indicated that a higher level of CFP could be expressed and secreted from *B. subtilis* containing different versions of the ChiSL promoter grown on the different media. However, the highest expression was observed in pUPChi-ICFP3 transformants grown in medium containing chitin (Fig. 5).

### Fluorimetric assay

**Fig. 6:**
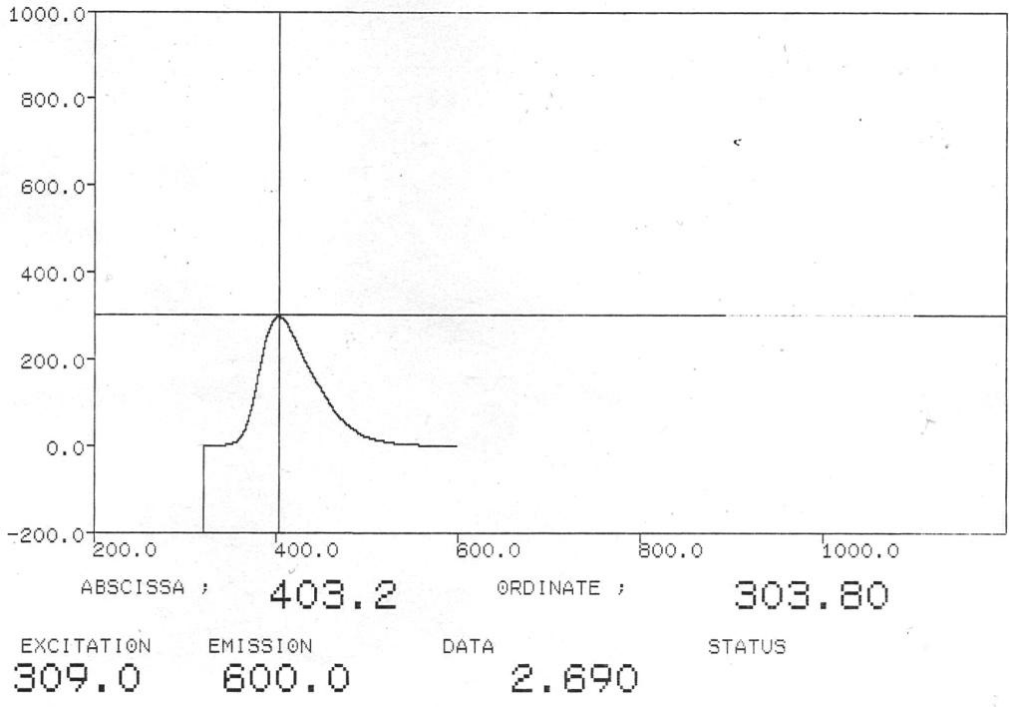
Florescent emission spectra from culture media of *B. subtilis* UpChi-CFP3. The horizontal and vertical axes represent the emission wavelength and relative fluorescence emission, respectively.

**Fig. 7:**
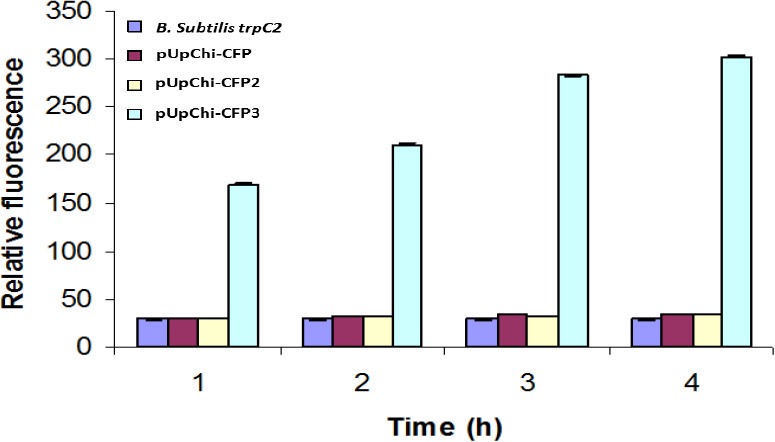
The fluorimetric assay for detection of relative promoter activity using ICFP, as a reporter protein. The activity of promoters fused to the ICFP reporter genes was detected using a “fluorescence spectrophotometer”. For each promoter, fluorescence was measured in quadruplicate, while each promoter was assayed at least in duplicate. Supernatant from the culture medium of *B. subtilis* trpC2 was used as a negative control.

The total fluorescence in extracellular protein extracts was determined using a fluorimeter to quantify the differences in the expression and secretion of CFP. Relative florescent emission of recombinant *B. subtilis* containing pUPChi-CFP, pUPChi-CFP2, and pUPChi-CFP3 was determined to be 12.99, 29.37, and 303.8, respectively. These results demonstrated that the production of ICFP in culture media consisting of chitin or chitin plus glucose is significantly higher in *B. subtilis* containing the UPChi-ICFP3 construct and is sufficient to be detected by the fluorometry as compared to the other promoter variants (Figs. 6 and 7).

## DISCUSSION

Promoter is a fragment of the DNA sequence, which drives the transcription of DNA to RNA[24]. Promoter strength plays a significant role in genetic engineering and biology. A constitutive promoter with a certain strength for a given RNA can often be reused for other RNAs[25]. Both theoretical and experimental methods can be used to evaluate promoter strength; however, there is a need to establish a system capable of assaying and comparing a promoter using promoter-reporter fusions.

GFP isolated from the jellyfish, *Aequorea victoria*, is a versatile reporter used in a wide variety of hosts in *in vivo* gene expression studies. It has also been shown that the mutagenesis of *gfp* results in variants with different fluorescent properties[26,27]. The CFP and YFP variants of GFP can be detected in cells directly without additives and substrates. This feature introduces them as a suitable marker for *in vivo* studies of multiple cellular processes within a single cell[28]. CFP can be applied as a molecular label or a reporter to visualize the position of a target protein inside living cells and its trafficking, as well as to quantify the protein expression. In addition to its application as a reporter gene, CFP and its derivatives are also used to monitor the introduction and expression of foreign genes in different cells and to evaluate the ability of transfecting materials to introduce plasmids or proteins into the cell[29].

Since the detection and screening of such transformants are cumbersome and time-consuming, the application of reporters such as luciferase and GFP, which allow transgene expression to be measured, greatly facilitate effective gene transfer technology[29].

GFP has been widely used in eukaryotes, especially in plant systems, for localization experiments and for gene transfer as a screenable marker[30-33]. Although used in plant and animal tissues with the same application as GFP, luciferase suffers from some disadvantages, including little toxicity or adverse effects on normal cell metabolism[34]. An important advantage of CFP and its optimized version, ICFP, is its suitability for consumption in prokaryotes, especially *Bacillus subtilis*[34]. ICFP is generated by an improvement and modification of CFP[16], making it possible to be used in a variety of gene transfer projects. In addition, this modified CFP, ICFP, has been shown higher fluorescence intensity in *Bacillus subtilis*. ICFP is widely used in prokaryotes compared to other fluorescent or luminescent markers[34]. Since its excitation and emission spectra are distinguishable from other fluorescent proteins, ICFP can be applied for numerous purposes such as co-localization of different proteins in the same cell simultaneously. ICFP is arbitrary for monitoring gene expression and protein displacement both *in vivo* and *in situ*[35-38].

The Gram-positive bacterium, *B. subtilis*, is among the best-studied microbial organisms displaying cellular differentiation. It has been extensively used as a model to study differentiating bacterial cell, secreting large quantities of proteins, and forming highly-resistant spores[39,40]. Hence, the availability of easily detectable ICFP in *B. subtilis* would remarkably facilitate the studies of promoter efficiency analysis in different organisms. The vectors for the production of fluorescent-protein fusions in *B. subtilis* containing the genes *cfp* and *yfp* have been studied previously[26]. However, these fusions, when expressed in *B. subtilis*, frequently display no or weak fluorescent signals. Herein, we showed that *icfp* is efficiently translated in *B. subtilis* using pUPChi-ICFP3. Furthermore, the chitinase promoter of *B. pumilus* SG2 and its derivative were used to evaluate the expression and secretion of ICFP. The full-length form was able to express ICFP at a very low level, which was undetectable by SDS-PAGE. However, the truncated version containing deletion in the CRE box and its upstream sequence in the pUPChi-CFP3 strain showed more effective expression and secretion of ICFP. Our present results revealed that inserting this truncated form of Chi promoter upstream of the ICFP, as a reporter gene, in *B. subtilis* led to an approximately tenfold increase in ICFP expression.
